# Empagliflozin and platelet-rich plasma improve stanozolol induced cardiotoxicity by reducing NF-κB/P65, IL-1B and apoptosis

**DOI:** 10.1186/s40360-026-01086-3

**Published:** 2026-02-23

**Authors:** Shaimaa M. Hafez, Eman Mohammed Elsaeed, Amal Hussain Mohammed Ali, Eman S. Said, Yara S. Abouelela, Asmaa Abdo Elshiech, Heba Abdelnaser Aboelsoud, Hala Magdy Anwer, Samar H. Elsharkawy, Marwa A. Ibrahim, Hamdy Rizk, Hoda A. Abd-Ellatieff

**Affiliations:** 1 Anatomy and Embryology Department, Faculty of Medicine for Girls, Al- Azhar University, Cairo, 11884 Egypt; 2 Anatomy and Embryology Department, Faculty of Medicine, Port Said University, Port Said, Egypt; 3 Department of Medical Laboratories, College of Applied Medical Sciences, Qassim University, Buraydah, 51452 Saudi Arabia; 4 Department of Medical Pharmacology, Faculty of Medicine, Fayoum University, Fayoum, 63511 Egypt; 5 Anatomy and Embryology Department, Faculty of Veterinary Medicine, Cairo University, Giza, Egypt; 6 Forensic Medicine and Clinical Toxicology Department, Faculty of Medicine for Girls, Al-Azhar University, Cairo, Egypt; 7 Department of Basic Medical Sciences, College of Medicine, Prince Sattam Bin Abdulaziz University, Al-kharj, Saudi Arabia; 8 Physiology Department, Faculty of Medicine, Benha University, Benha city, Egypt; 9 Department of Surgery, Radiology and Anesthesiology, Faculty of Veterinary Medicine, Cairo University, Giza, Egypt; 10 Biochemistry and Molecular Biology Department, Faculty of Veterinary Medicine, Cairo University, Giza, Egypt; 11 Department of Pathology, Faculty of Veterinary Medicine, Damanhour University, El-Beheira, Egypt; 12 Department of Pharmacology and Toxicology, College of Pharmacy, Qassim University, Buraydah, 51452 Saudi Arabia

**Keywords:** Cardiotoxicity, Pathology, Apoptosis, Stano, EP, PRP

## Abstract

**Background:**

Stano is an anabolic androgenic steroid (AAS) with various adverse cardiovascular effects because it induces ventricular dysfunction. The objective of this study was to evaluate and demonstrate how Empagliflozin (EP) and platelet-rich plasma (PRP) can reduce the cardiotoxic effects linked to Stano administration in albino rats.

**Methods:**

Fifty-four (*n* = 54) rats were involved in this research, the animals were randomly divided into six groups: (C, EP, PRP, Stano, SEP, and SPRP). Various cardiovascular health markers, focusing on the relationship between oxidative stress and inflammation in cardiomyopathy were evaluated.

**Results:**

There was extensive myocardial injury and damage in the Stano group, as reflected by induction of oxidative stress by increase in the level of MDA, along with significantly reduced level of GSH and CAT. Increased expression of inflammatory markers like IL-1β, and NF-κB/P65 pathway, with strong immunoreactivity of caspase-3-an apoptosis marker in cardiac tissue were observed. Evidence of left ventricular hypertrophy with pathological changes in cardiac tissues was also noted, together with functional impairments. Hypertrophy acts as a compensatory mechanism against injury and stress, since it may cause various complications in case of a lack of intervention.

**Conclusion:**

Stano activates the initiation of some apoptotic and inflammatory signaling pathways to cause serious aggravated cardiovascular health threats. On the other hand, all the structural function and antioxidant parameters related to cardiovascular health showed improvement after both EP and PRP treatments. Intriguingly, EP and PRP have emerged as effective promising agents against the deleterious cardiac damage linked to Stano use. Nevertheless, more research is actually needed to explain the mechanisms and optimize clinical application or management of those exposed to ASS.

## Introduction

 Anabolic androgenic steroids (AAS) are man-made analogs of testosterone, and its main use is to increase body function through the development of muscular mass. Because they tend to attach predominantly to the androgen receptors in cells, causing many physiological chains such as increased muscle strength and size [1]. They increase and promote the creation of the protein and relieve the soreness that results from overworkouts and hence are preferred by bodybuilders and sportsmen [2]. There are two classes of anabolic steroids available, two types of derivatives, the first is 17 α alkyls, including oxandrolone, Stano, and fluoxymesterone; the second is 17 ß esters, including testosterone cypionate and testosterone enanthate [3, 4]. The abuse of the use of AAS among athletes and bodybuilders is a major issue, specifically, since the drugs are increasingly available and easily accessible via various marketing campaigns, including the Internet [5].The abuse of AAS is increasing due to the demand for improved physical performance and appearance [6] despite Cs by the World Anti-Doping Agency (WADA) agencies. In the United Kingdom, they are classified as Class C drugs, making their possession and sale illegal without a medical prescription, however, they can be purchased from any place without a medical prescription in Egypt [7]. The use of this ASS in either legal or illegal form is increasing [1], however, its use can produce unwanted side effects such as endocrine imbalance and psychiatric issues in the form of anxiety and depression when withdrawn [2]. In addition, AAS are linked with several serious cardiovascular adverse effects including increased cardiac contractility and relaxation [8], arrhythmias [9], hypertension [10], atherosclerosis [11], ventricular dysfunction [12], and sudden death [13]. Fibrosis, or the deposition of collagen, is a result of heart failure development and causes extracellular matrix remodeling [14].

Empagliflozin (EP), a sodium-glucose co-transporter 2 (SGLT2) inhibitor, is primarily employed for blood glucose C in patients with type 2 diabetes, apart from its use in induction of massive natriuresis, particularly when combined with loop diuretics, leading to increased blood volume without causing electrolyte disturbance or neurohormonal stimulation [15]. Its use goes very deep into cardiovascular health, particularly among patients with heart failure [16]. EP possesses a large promise to decrease the risk of hospitalization due to heart failure by lowering the cardiovascular mortality of the patient with both preserved and reduced ejection fractions [17]. The EP has managed to decrease the occurrence rate of severe heart failure events in more than 40% of individuals with a left ventricular ejection fraction (LVEF). This benefit is also observed in patients with atrial fibrillation or flutter cardiovascular diseases [18]. It is associated with reductions in blood pressure and body weight without raising the heart rate, which makes it effective and helpful for patients with more than one medical condition [19]. Besides its role in reducing glycaemia, EP is also capable of offering protection against cardiotoxicity and injury from certain chemotherapy drugs, including doxorubicin because it can reverse doxorubicin-induced left ventricular dysfunction and remodeling [20].

Platelets act as the first responders to trauma and damage to the body, activating and aggregating quickly at the site of injury. Once activated, they play a crucial role in inflammation regulation and managing tissue remodeling, particularly via the action of interleukin-1beta (IL-1β) [21]. Upon tissue injury, platelets effectively halt bleeding through the formation of a fibrin clot, this initial aggregation besides preventing blood loss, also creates and aids in the formation of a framework for subsequent healing processes. In addition, releasing of various signaling molecules attracts additional immune cells to the injury site upon activation of the platelets [22]. Activated platelets secrete multiple growth factors and inflammatory cytokines that significantly impact both tissue regeneration and inflammation. Furthermore, platelets are essential agents for angiogenesis which is crucial for delivering oxygen and other nutrients to the healing tissues [22, 23].

Several and multiple innovative platelet-based products, such as platelet-rich fibrin, bone marrow aspirate/concentrate, adipose tissue, and platelet-rich plasma (PRP) [24], have been developed and emerged for clinical use in the transfusion medicine field, as they promote tissue repair and wound healing. These products can be allogeneic or autologous as they are abundant in cellular and molecular elements [25]. Additionally, PRP, also known as platelet concentrate, is an autologous derivative of whole blood with a greater platelet count than peripheral blood [26]. PRP has shown a beneficial role in various medical fields, particularly in treating joint injuries, osteoarthritis, and other musculoskeletal conditions [25]. Furthermore, they promote tissue granulation and angiogenesis by releasing many growth factors such as EGF (Epidermal Growth Factor), VEGF (Vascular Endothelial Growth Factor), and PDGF (Platelet-Derived Growth Factor) [27]. Recent research by El-Shafei et al. [28] indicates that PRP can significantly alleviate heart damage caused by chemotherapy agents like cisplatin. The objective of this study was to evaluate and demonstrate how EP and PRP can reduce the cardiotoxic effects linked to Stano administration in albino rats.

## Methods

### Reagents and chemicals

The Stano ampoules containing a 50 mg/ml Stano suspension (Stromba Aqua) from (Spectrum Pharmaceuticals) and the EP (10 mg tablets of EP (EPcoza) obtained from Zeta Pharma were used in our study. Additionally, phosphate-buffered saline (PBS) and citrate dextrose from (Sigma-Aldrich), along with xylazine (sourced from Xyla-Ject^®^ at a concentration of 2%) were purchased.

### Animal management and ethical approval

The Institutional Animal Care and Use Committee of Cairo University/Faculty of Veterinary Medicine (Vet-CU- IACUC) approved this investigation to be carried out. The ethical approval is identified by the (Vet-CU- 03162023676) number, confirming that the research adheres to regulations and ethical guidelines for animal handling and management.

A total of 60 adult Sprague-Dawley albino rats, each weighing between 180 and 200 g were sourced from the (Egyptian Organization of Biological Products and Vaccines/ Cairo, Egypt) and involved in this study. A number of six animals (*n* = 6) from these Sprague-Dawley albino rats were utilized to create the PRP using their blood samples.

The rats were kept in a well-ventilated stainless-steel cage that measured 37 × 38 × 17 centimeters at room temperature. They were adapted to a 12-hour light/dark cycle and had unrestricted access to standard rodent pellets and fresh water. The rats were allowed a week to acclimate to their new surroundings after arrival, offered with both institutional and national animal care standards. The research was conducted at the Faculty of Medicine for Girls at Al-Azhar University in Cairo Animal House. Strict hygienic measurements for care throughout the study were followed to maintain a healthy environment for the experimental animals.

### Procedures for preparing and activating PRP

Rats are sedated with xylazine at a dose of 1 mg/kg I/M [29]. Using a capillary tube and a citrate dextrose solution as an anticoagulant with a 1:9 citrate/blood ratio, two milliliters of blood were extracted from the retro-orbital plexus in an aseptic manner [30, 31]. Following three minutes of spinning at 3000 rpm for the blood, the plasma, and buffy coat supernatant were collected and centrifuged again at 4000 rpm for fifteen minutes in a fresh tube. Next, we removed the upper two-thirds, which included the platelet-poor plasma and kept the lower third, which was created by PRP. After that, the PRP was gathered in a tube holding 0.4 milliliters of PBS. Including 2.4 × 10^6^ platelets/ml, the final fraction was approximately 3.9 times higher than the blood platelet count of 580 000 platelets/ml. With every injection, new and fresh PRP was administered [32]. Finally, right before injection, the PRP was activated with 10% CaCl₂ (0.4 ml of PRP + 0.1 ml of CaCl₂), as per Hafez et al.‘s 2021 study. For four weeks, rats received subcutaneous injections of 0.5 ml/kg PRP (containing 0.2 ml pure PRP) at the interscapular region using a sterile insulin syringe, twice a week [33, 34].

### Experimental design

The study involves six experimental groups of nine rats each. The first group is the negative group (C, G1), administered 4 ml of distilled water (DW) orally via gastric gavage for four weeks (wks). The next group is the EP (G2), who received EMPA at a dosage of 10 mg/kg/day, delivered orally in 4 ml of DW through gastric gavage for four wks [35–38]. The PRP, (G3), was administered 0.5 ml/kg of PRP, consisting of 0.2 ml of pure PRP, subcutaneously twice a week for four wks [34]. The Stano (G4), was given 3.6 mg/rat/day of Stano subcutaneously for four wks [39]. The SEP (G5), received both S and EP in the same dosages and time frames as previously described. The 6th group (SPRP, G6), received the S and PRP as detailed in G3. The animals were monitored for clinical signs at least 3 times daily.

At the end of the experiment (on the day 29th from the experiment), all the experimental rats were euthanized with an intraperitoneal pentobarbital injection (45 mg/kg, i.p) [40, 41].

### Evaluations of echocardiography

Our all rats were pre-trained and manually restrained by a skilled handler for echocardiography. The echocardiographic acquisition time was limited to 2–3 min per animal to reduce handling stress. This approach was selected to avoid the known depressant effects of anesthetic agents on cardiac function and to ensure accurate physiological measurements [42]. The offline measurements are then taken afterwards [43, 44]. Briefly, following depilation of the rats, a warm ultrasound gel was administered. A 13-MHz phased array transducer was used in the Aloka F31 machine (Hitachi Corporation, Japan) to perform baseline transthoracic echocardiography. All cardiac data were acquired from the M mode graphs from short axis views [45]. As previously mentioned, measurements were averaged from three consecutive cardiac cycles at both end diastole and systole. These measurements included LV posterior wall (LVW), LV internal diameter (LVID), and interventricular septal thickness (IVS) [42], computing the ejection fraction and fractional shortening (FS%), which are utilized to assess systolic function in accordance with earlier formulations [42]. Similarly, all groups underwent re-examinations following a 4-week S induction period and following EP and PRP treatment. As a result, all measurements and indices were computed using the prior values.

### Cardiac serum marker assay

Creatine kinase-myoglobin binding (CK-MB) and lactate dehydrogenase (LDH) activities were assessed using colorimetric kits sourced from HUMAN Diagnostics in Wiesbaden, Germany.

### Oxidative stress and antioxidant activity assay

The oxidative stress biomarkers, including Malondialdehyde (MDA), reduced glutathione (GSH), and important antioxidant enzymes such as Catalase (CAT) were measured using cardiac homogenates following the established protocols from manufacturers like Laboratory Biodiagnostics in Cairo, Egypt.

### Histopathology

Following the acquisition of cardiac tissue, a part of the cardiac tissue samples was then fixed in 10% neutral buffered formalin (10% NBF). For histopathological examinations, the tissues underwent dehydration through a series of ethanol solutions, gradually removing moisture to prepare them for embedding. After dehydration, the samples were cleared using xylene, and the cleared samples were embedded in paraffin wax. Paraffin blocks were then sectioned with a microtome to achieve slices of 4–5 μm thickness. The sections were stained using hematoxylin and eosin (H&E), and the H&E-stained sections were examined under a microscope, specifically a Leica microscope (Leica Microsystems, Switzerland). For histological assessment, samples were examined using a digital camera integrated imaging system (DM300, Leica, Germany). The microscopic lesions were classified into four grades based on their severity as (0), no lesions observed; (1), mild lesions present, affecting less than 10% of the heart tissue; (2), moderate lesions score affecting between 10% and 50% of the heart tissue. The 3rd grade (3), represents severe lesions impacting more than 50% of the heart tissue [46–49].

### Collagen fibers estimation using red stain Sirius

Heart sections were stained with Sirius Red stain (Sigma-Aldrich, “Direct Red 80,” Cat. # 36-554-8, USA) to precisely visualize the collagen fibers. The slides were first deparaffinized and then rehydrated. After that, they were rinsed in distilled water and submerged in Weigert’s hematoxylin solution for five minutes to stain the nuclei. Subsequently, the slides were immersed in a Picro-Sirius red stain for a minimum of 60 minutes, followed by two rounds of washing in 0.5% acetic acid water. They were then rapidly dehydrated in absolute alcohol (three changes), cleaned with xylene, and covered with a coverslip [50]. Six portions of the Image Analysis System (L.A.S.) were used for the quantitative measurement of collagen fibers. [Leica Microsystems, Cambridge, UK] “software v.4”.

### Immunohistochemistry analysis

Immunohistochemical labeling staining was performed on all heart tissues examined histologically using the avidin-biotin complex (ABC) technique using ABC kits (Vectastain ABC-HRP kit, Vector Labs). The following antibodies have been tested: Rabbit NFκB-p65 Polyclonal Antibody (Elabscience Cat# E-AB-32232, Dilution: 1:100), Rabbit Anti-active Caspase 3 Polyclonal Antibody, Unconjugated (Abcam Cat# ab13847, RRID: AB_443014), and Rabbit Anti-IL-1β antibody, Clone [RM1009] (Abcam Cat# ab283818, Dilution: 1:500). After adding the secondary antibody (biotinylated anti-rabbit IgG; DAKO Cytomation, Carpinteria, California), the bound labels peroxidase was visualized with the liquid DAB substrate chromogen system (DAB, Sigma). Sections were further counterstained with Meyer’s hematoxylin [51, 52]. Negative Cs are integrated using non-immune serum rather than primary or secondary antibodies. A Leica microscope (CH9435 Hee56rbrugg) with various magnification powers was used to examine and take pictures of the immunostained sections (Leica Microsystems, Switzerland).

### Quantitative scoring for immunohistochemistry results “Area Percentage”

Six fields with high power (x 400) positive brown immunostaining spots were chosen for assessment in each serial segment of the groups under study. Area percentage was calculated using the Leica QWin 500 image analyzer computer system (England) for sections stained with Caspase 3, IL1β, and NF/kB-P65. A Leica microscope with a colored video camera, a colored monitor, and the hard drive of a Leica IBM personal computer connected to the microscope and Cled by Leica QWin 500 software was needed for this image analyzer.

### Analytical statistics

The Kolmogorov-Smirnov test of normalcy was used to determine the normalcy of all the parameters and data. According to the test results, the majority of the data had a normal distribution (parametric data), which led to descriptive analysis. Testing for intergroup relationships was done using One Way-ANOVA and the Post-Hock tests. P-values were considered statistically significant if they were less than 0.05. To conduct the statistical analysis, the SPSS 26.0 (Statistical Package for Scientific Studies, SPSS, Inc., Chicago, IL, USA) program for Windows was used.

## Results

### Echocardiographic findings

**Fig. 1:**
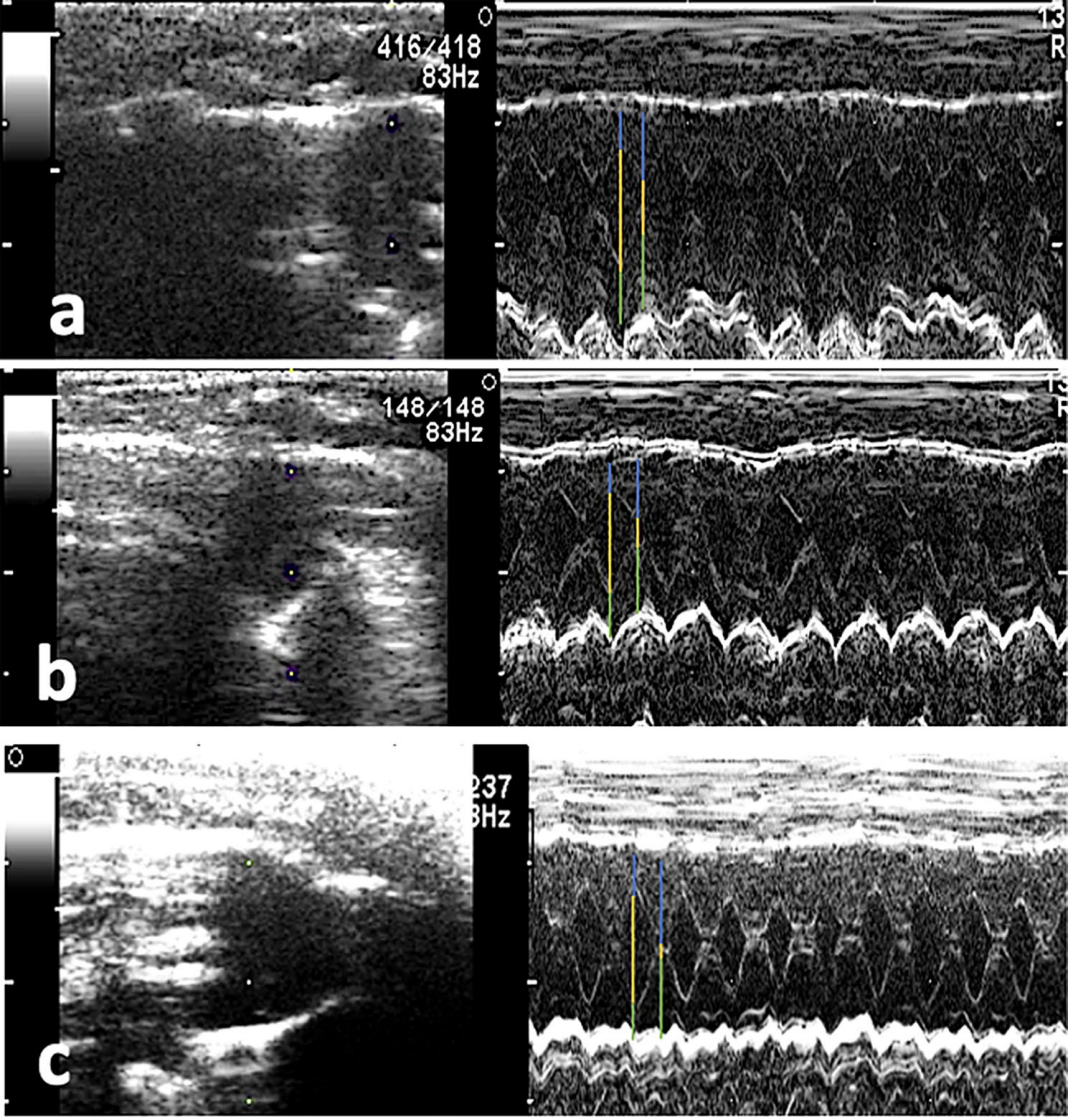

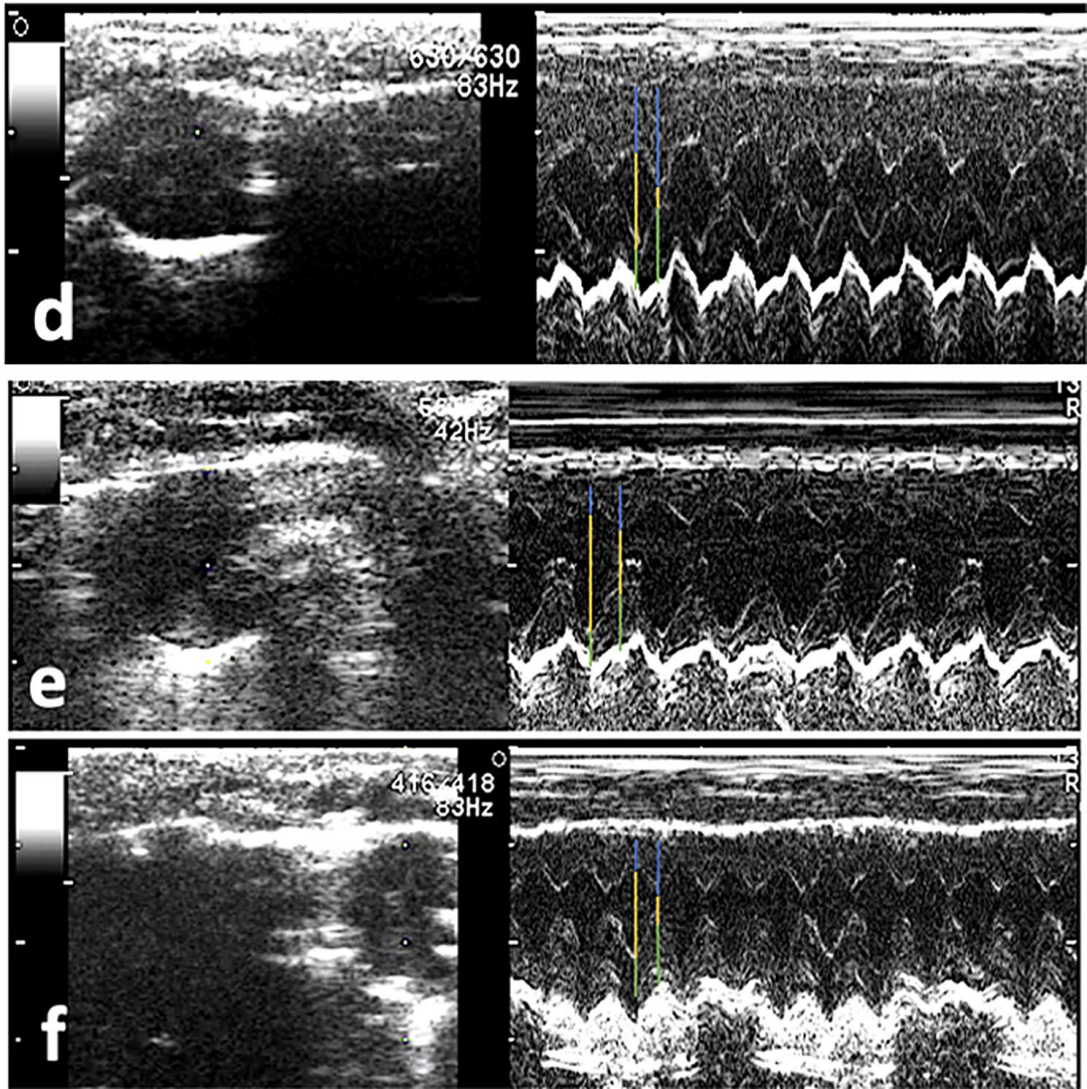
Photomicrographs demonstrating M- mode echocardiography of the left ventricle for different groups, Images (**a**) and (**b**) show normal cardiac dimensions at baseline examination including interventricular septal thickness (blue lines), left ventricular internal dimensions (yellow lines) and left ventricular free wall (green lines) at both end diastole and systole respectively. After receiving Stano, (**c**) and (**d**), both interventricular septal thickness and left ventricular free wall were hypertrophied with reduction of the left ventricular internal dimensions at end diastole and systole respectively. SEP and SPRP treated groups (**e** and **f** respectively) showed complete resolution of the left ventricular hypertrophy

All echocardiographic measurements are summarized in Table 1. The baseline groups (C) (Fig. 1) showed normal interventricular septal thickness, left ventricular internal dimensions and left ventricular free wall at both end diastole and systole. In groups treated with Stano, there was a significant hypertrophy of the IVD and IVS with significant reduction of the LVID and LVIS compared to the baseline group. There was no significant difference between the baseline (C) and both SEP, and SPRP treated groups except for the LVID and LVIS which was higher in the treated groups. In the SEP, there was a significant reduction of the IVS with increased LVID than the S group (Fig. 1). The IVD, LVID and LVIS showed significant improvement in the SPRP than the C group. The systolic function as indicated by FS % was significantly reduced in the Stano group than the C and treated groups. The EF% showed no difference between the groups.

**Table 1:** Echocardiographic measurements and indices for all groups at baseline (C), after induction by Stano and treated groups with EP and PRP

	Baseline	Induction	Treatment			*P* value
	Control	Stano	SEP	SPRP		
IVD	1.19 ± 0.14 ^b^	2.03 ± 0.32 ^a^	1.61 ± 0.28^ab^	1.35 ± 0.08^b^		< 0.001
	1.02–1.41	1.46–2.50				
LVID	5.4 ± 0.12 ^b^	4.23 ± 0.50 ^c^	6.30 ± 0.28^a^	5.5 ± 0.14^a^		< 0.001
	5.20–5.50	3.80–5.26				
LWD	1.93 ± 0.35	2.17 ± 0.43	2.04 ± 0.52	1.65 ± 0.47		0.371
	1.50–2.37	1.75–2.95				
IVS	2.34 ± 0.29 ^b^	3.07 ± 0.28 ^a^	2.48 ± 0.09^b^	2.31 ± 0.4^ab^		< 0.001
	1.95–2.81	2.63–3.49				
LVIS	2.59 ± 0.16 ^b^	1.86 ± 0.17 ^a^	3.45 ± 0.35^ab^	2.55 ± 0.07^b^		< 0.001
	2.40–2.80	1.53- 2.00				
LWS	2.52 ± 0.31	2.75 ± 0.56	2.66 ± 0.40	2.22 ± 1.03		0.542
	2.08–3.01	1.74–3.57				
FS %	51.98 ± 2.77^a^	55.53 ± 6.89 ^a^	45.31 ± 3.16^b^	53.60 ± 2.48 ^a^		0.03
	48.15–56.36	47.37–67.31				
EF %	87.63 ± 2.03	83.91 ± 12.19	85.64 ± 8.12	81.75 ± 3.18		0.701
	84.70–90.70	55.20–93.70	79.50–90.00	89.45 ± 0.78		

* Different superscripts show significant difference between the groups at *p* ≤ 0.05 using One-way ANOVA and post hoc Tukey’s test. EP; Empagliflozin, PRP; platelet rich plasma, IVD; interventricular septum at end diastole, LVID; left ventricular internal diameter at end diastole, LWD; left ventricular free wall at end diastole, IVS; interventricular septum at end systole, LVIS; left ventricular internal diameter at end systole, LWS; left ventricular free wall at end systole, FS; fractional shortening, EF; ejection fraction. Values expressed as Mean ± SD, Different superscripts (a, b, and c) indicate significant difference at *p*-value ≤ 0.001

### Myocardial injury indicator

The Stano-treated rats (G4) exhibited significantly higher levels of CK-MB and LDH concerning the NC (G1). On the contrary, the S treated groups either with EP (SEP, G5), or PRP (SPRP, G6) displayed a notable decline in the cardiac serum levels of CK-MB and LDH. Noticeably, the SPRP (G6) dramatically decreased the CK-MB level compared to the Stano-treated group (G4), (Table 2). The rats received PRP (G2) and EP (G3) showed normal levels of CK-MB relative to the G1.

**Table 2:** Results of the CK-MB and LDH in the serum

	Control	EP	PRP	Stano	SEP	SPRP
CK-MB (U/L)	120.4±4.1e	119.8±1.4e	122.1±2.2d	248.5±4.1a	155.3±0.87c	174.2±2.1b
LDH (U/L)	1600±0.01e	1605±0.03e	1480±0.01d	3850±0.01a	2200±0.02bc	2300±0.03b

Mean values of serum CK-MB, and LDH are significantly different at P ≤ 0.05 using ANOVA test. Data are presented as (Mean ± SEM), SEM = Standard error of mean

### Oxidative and antioxidant markers assay

**Fig. 2:**
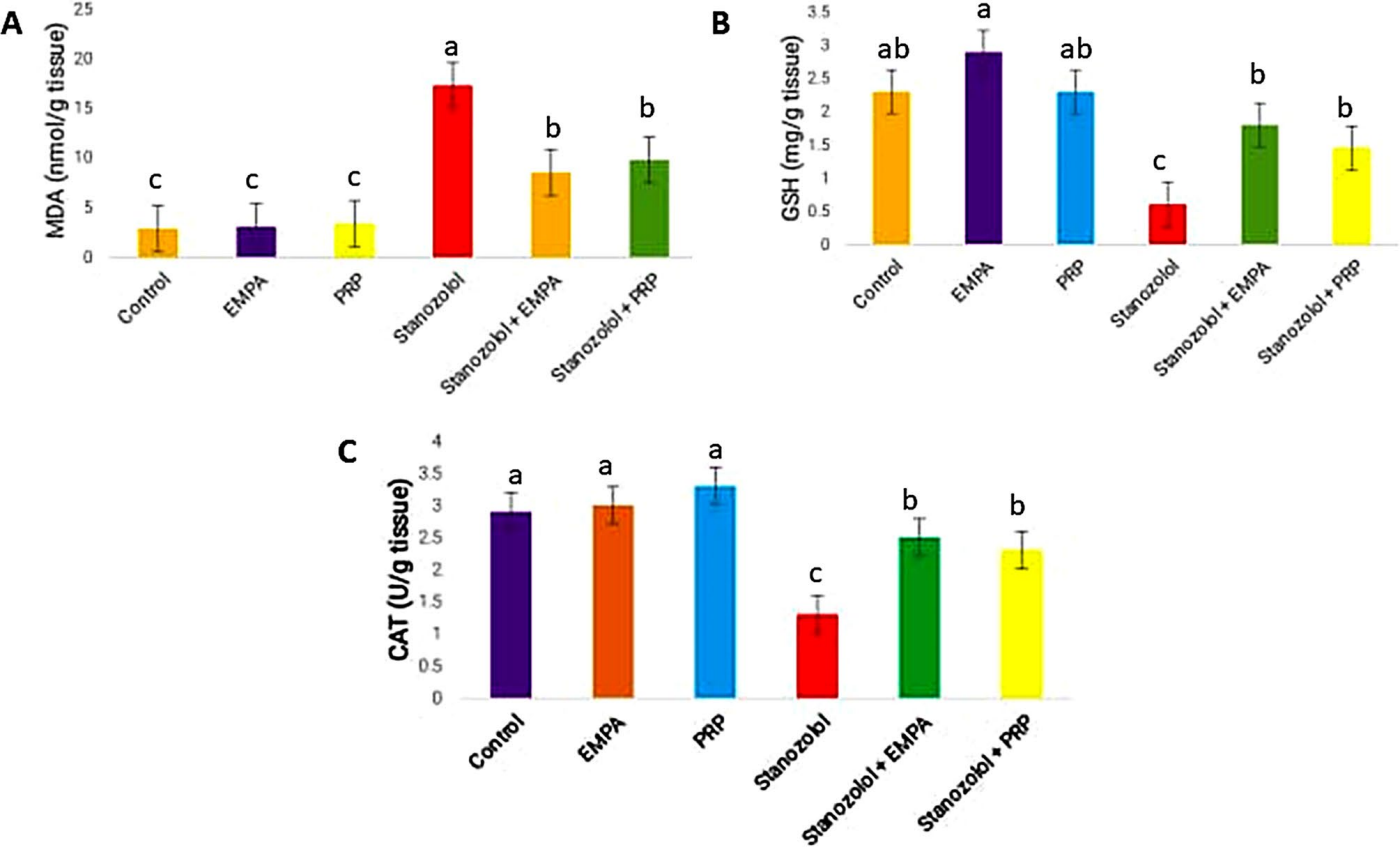
Mean values of cardiac tissue MDA, GSH, and CAT in different experimental groups that are significantly different at (*P* ≤ 0.05). Data are presented as (Mean ± SEM), SEM = Standard error of the mean. Different superscripts (**a**, **b**, and **c**) indicate significant difference at p-value ≤ 0.001

Stano triggered substantial oxidative stress injury in G4 as depicted in Fig. 2, and Table 2. A noticeable increase in the MDA level accompanied by a remarkable decrease in the CAT and GSH levels was detected in the G4. Notably, the oxidative injury induced by Stano was considerably reduced in SEP, and SPRP, as illustrated by a substantial reduction in the MDA level and subsequent increase in CAT and GSH indices.

### Cardiac histopathology and fibrosis

**Fig. 3:**
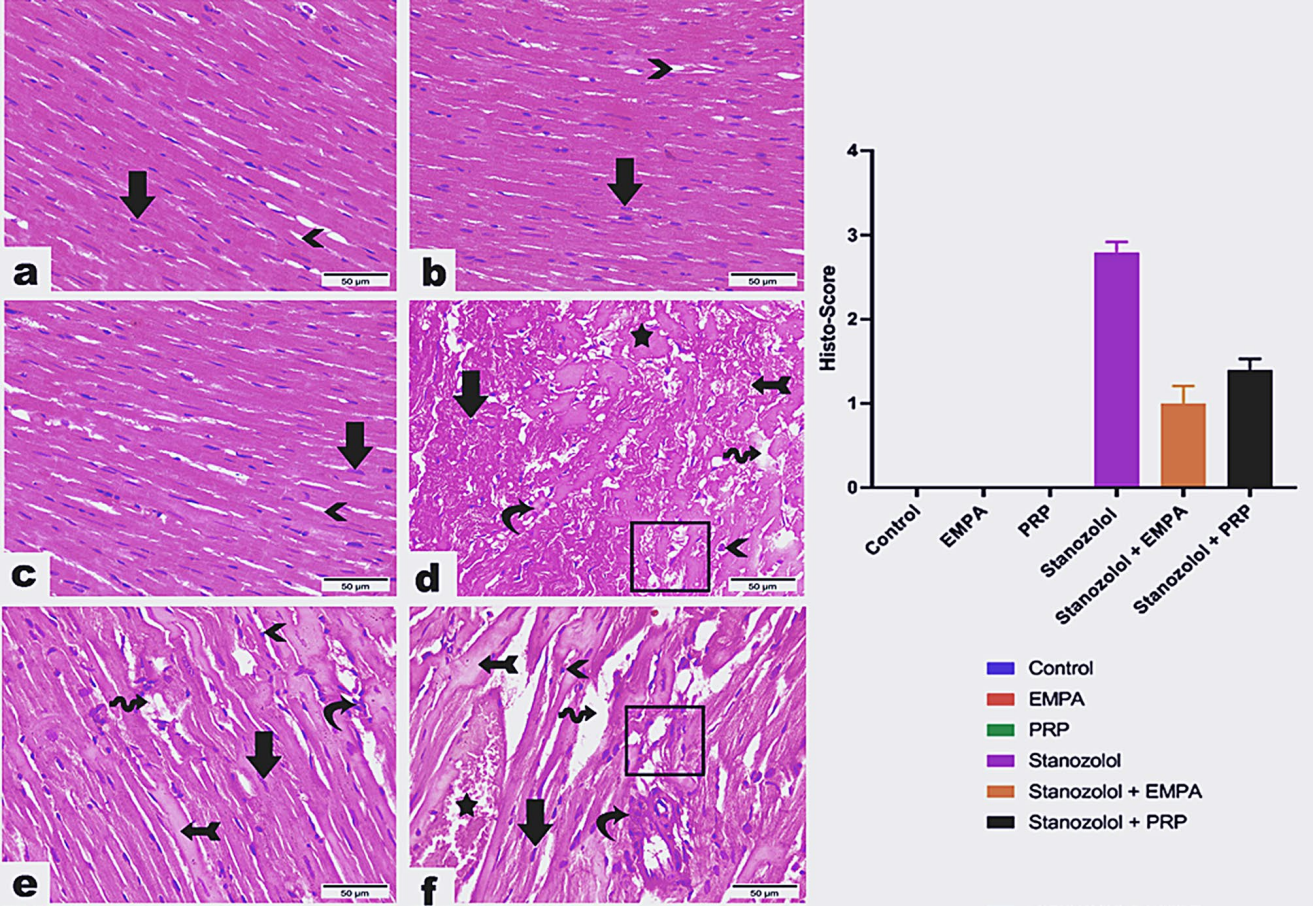
Photomicrographs displayed the histopathological alterations along heart tissue sections between inspected groups (Hematoxylin & Eosin Stain, Magnification Power = x400 & Scale Bar = 50 μm): Sections from C group (**a**), EP group (**b**), & PRP group (**c**) signifying the standard histological architecture of cardiomyocytes. They seem elongated, branched, and cross striated with large oval central nuclei (arrow) and narrow slit-like interstices (arrowhead) in between. (**d**) Stano group highlighting severe histological alterations including degenerated cardiac myofibers with loss of its organization (rectangle), focal hypertrophy with necrotic areas (star), and cardiac amyloidosis (curvy arrow). Moreover, cardiac myocytes marked either in hydropic degeneration form (arrowhead) or deep basophilic apoptotic ones (arrow). Additionally, interstitial fibrosis (arrow with tail) as well as edema (wave arrow) leading to dispersion between cardiac myofibers. (**e**) SEP group exhibiting an obvious improvement along heart tissue evidenced by almost myofibers detected with apparently normal cardiomyocytes (arrow) except few ones with deep basophilic apoptotic nucleus (arrowhead), and few myofibers still noticed in hypertrophied form (arrow with tail). Few interstitial infiltration of inflammatory cells (curvy arrow) and limited areas with interstitial edema (wave arrow) were also noticed. (**f**) SPRP group demonstrating moderate development along heart tissue manifested by focal areas with loss of their organization (rectangle), some myofibers spotted as normal cardiomyocytes (arrow) and other ones with deep basophilic apoptotic nucleus (arrowhead), and some myofibers still noticed in hypertrophied form (arrow with tail). Moreover, marked dilatation and congestion of blood vessels (star), moderate infiltration of inflammatory cells (curvy arrow) as well as interstitial edema (wave arrow) were also noticed. Histo-score; Values expressed as Mean ± SD, Different superscripts (**a**, **b**, and **c**) indicate significant difference at *p*-value ≤ 0.001

Elongated, branched, and cross-striated myocardial muscles with obvious large oval central nuclei and narrow slit-like interstices in between were seen in the histopathological changes of the heart tissues of rats from the C, EP, and PRP experimental groups G1, 2, and 3, respectively. These structures did not show any pathological changes (Fig. 3a, b, and c). However, a histological examination of the heart tissue from group G4 that was given Stano showed important histopathological changes, such as focal necrosis with disorganized cardiac fibers, focal hypertrophy in a lot of cardiac myofibers, and degeneration of myocardial muscles. All of the rats under examination also have cardiac amyloidosis. Additionally, there was noticeable hydropic degeneration of the heart muscles, along with profound basophilic apoptotic cells. Furthermore, nearly all portions showed interstitial fibrosis and edema that caused cardiac myofiber dispersion (Fig. 3d). On the other hand, the group that underwent EP treatment following Stano administration demonstrated a significant improvement in the heart tissue, as evidenced by the identification of cardiomyocytes that appeared normal and exhibited normal myofiber arrangement. The tissues under study displayed a few hypertrophied myofibers, deep basophilic apoptotic nuclei, minimal inflammatory cell infiltrations, and minor interstitial edema (Fig. 3e). After receiving S treatment, rats in SPRP (G6) showed modest interstitial edema and a moderate inflammatory response. The rats’ heart tissues showed a moderate degree of improvement in the experiment, as evidenced by normal cardiomyocytes and a few deep basophilic apoptotic nuclei in certain cardiac muscles, as well as specific areas of myocardial necrosis. Additionally, in a few areas, there was noticeable blood vessel dilatation and congestion (Fig. 3f).

**Fig. 4:**
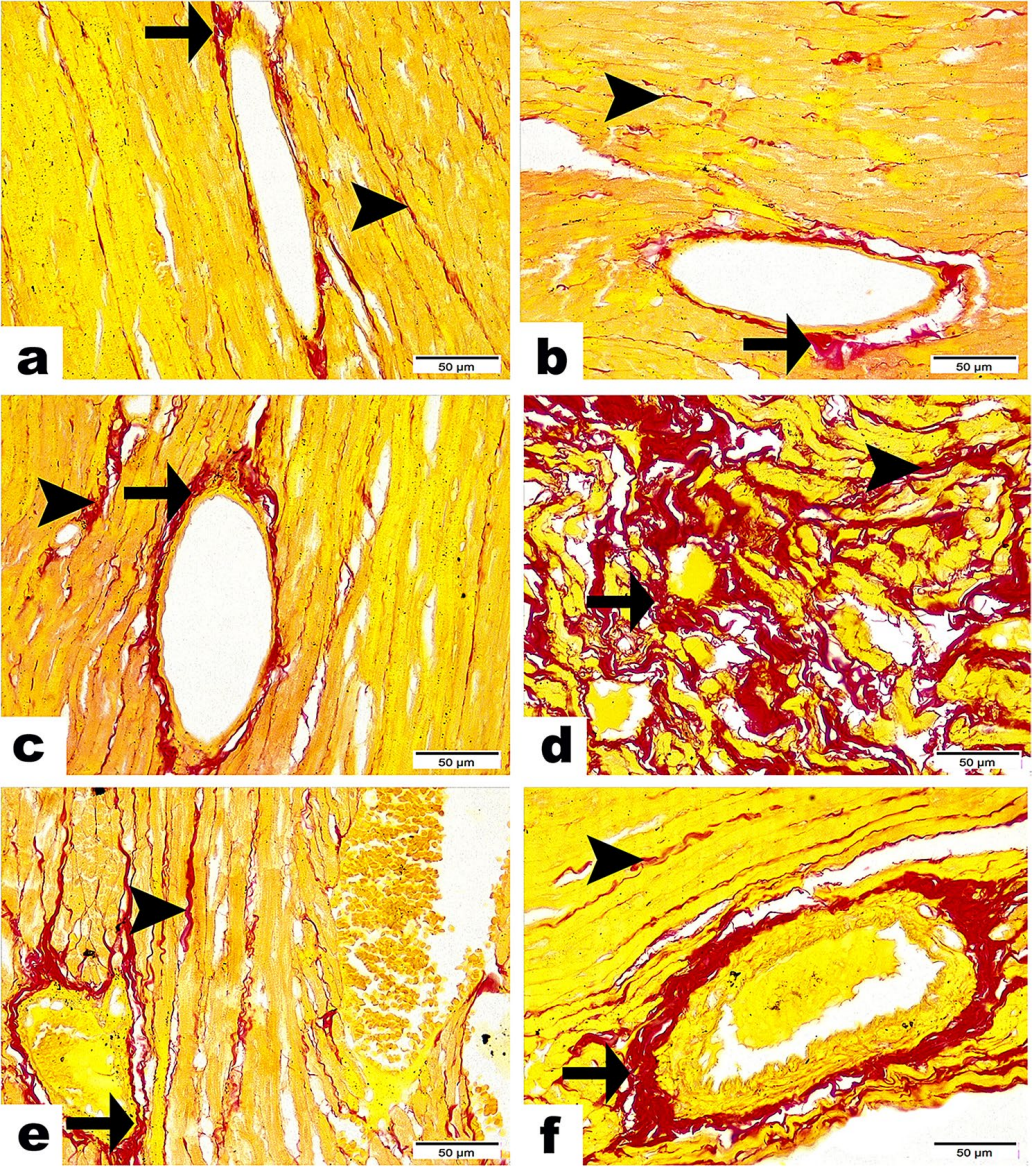
Photomicrographs presented the amount of collagen fibers in red color along heart tissue among studied groups (Sirus Red Stain). Sections from C group (**a**), EP group (**b**), & PRP group (**c**) revealing scarce amount of collagen fibers encircling blood vessels (arrows) as well as in between cardiac myofibers (arrowhead) with non-significance between them. (**d**) Stano group representing the highest amount of collagen fibers surrounding blood vessels (arrow) as well as in between cardiac myofibers (arrowhead) with significant difference (*P* ≤ 0.001) from C, EP, & PRP groups. (**e**) SEP group marking few quantities of collagen fibers surrounding blood vessels (arrow) as well as in between cardiac myofibers (arrowhead) with significant difference (*P* ≤ 0.001) from C, EP, PRP & Stano groups. (**f**) SPRP group showing moderate amounts of collagen fibers surrounding blood vessels (arrow) as well as in between cardiac myofibers (arrowhead) with significant difference (*P* ≤ 0.001) from C, EP, PRP, SEP and SPRP groups. Scale Bar = 50 μm

Regarding the quantity of collagen fibers that were found using the Picro-Sirius red stain, the G1 (Fig. 4a), G2 (Fig. 4b), and G3 (Fig. 4c) showed a small number of collagen fibers that were not significant in size surrounding blood vessels and in between cardiac myofibers. However, compared to the C groups, the Stano-treated group (Fig. 4d) showed a statistically significant increase in the amount of collagen fibers surrounding blood vessels and in between cardiac myofibers. Both the PRPS and EPS treated groups (Fig. 4f and e) had significantly less collagen fibers surrounding blood vessels and in between cardiac myofibers. Noting the EPS group’s expression of the lowest amount of collagen fiber deposition (Fig. 4; Table 3).

**Table 3:** Results of quantity of collagen fibers using sirus red stain

	Control	EP	PRP	Stano	SEP	SPRP
**Collagen Fibers**	1.243 ± 0.158	1.765 ± 0.135	1.6127 ± 0.36	34.56 ± 1.319a	5.584 ± 0.544c	11.978 ± 0.287 b
**F- Value =** 2640.370 ***P*- Value =** 0.000						

Values expressed as Mean ± SD (Area %). Different superscripts (a, b and c indicate significant difference at *p*-value ≤ 0.001

### Immunohistochemistry expression of IL-1β and NF-κB/P65 in the cardiac tissue

**Fig. 5:**
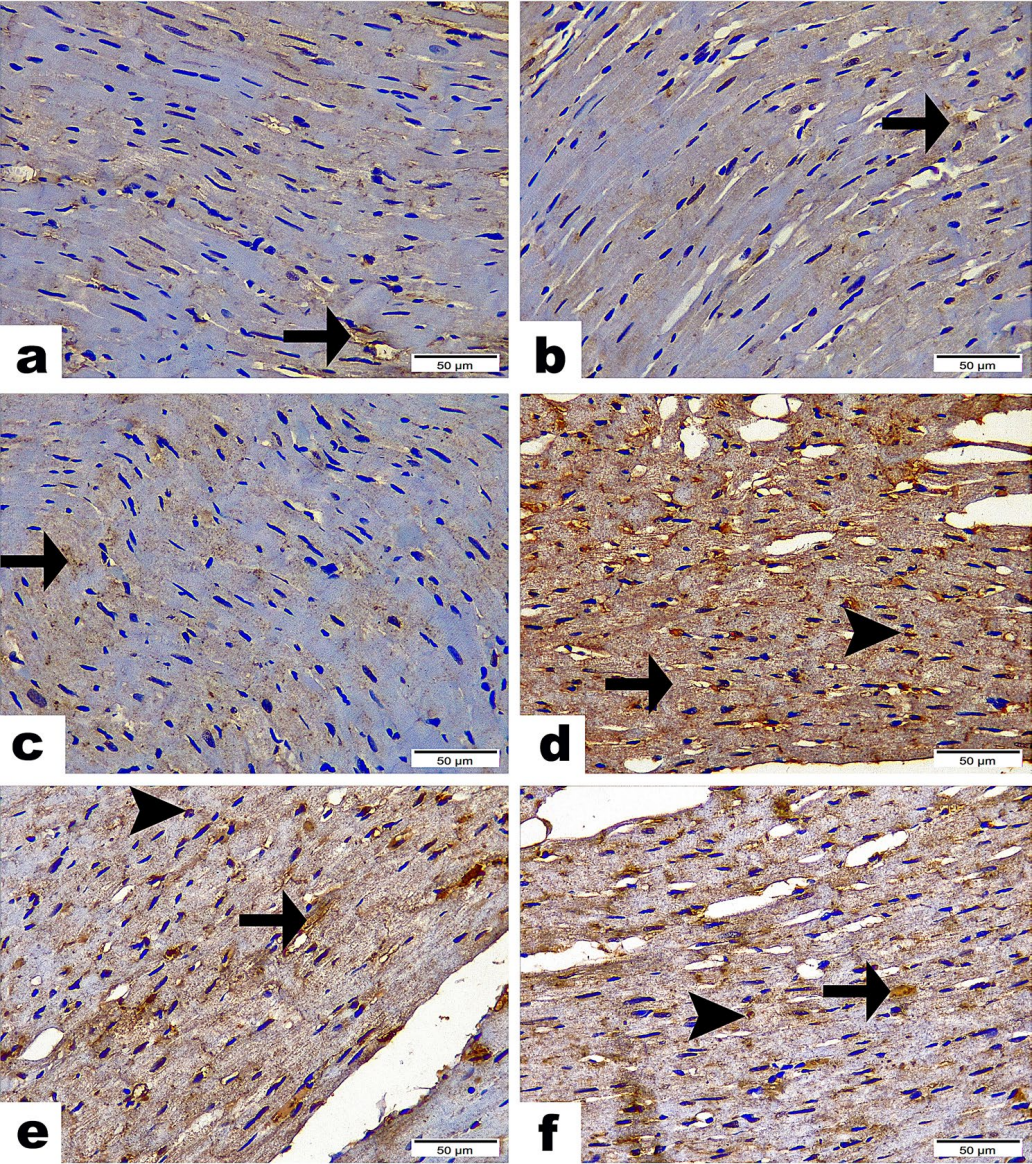
Photomicrographs exhibited the reactivity of IL1β along heart tissue sections between experimental groups. Sections from C group (**a**), EP group (**b**), & PRP group (**c**) showing scarce reactivity of IL1β along cardiac myofibers (arrows) with non-significance between them. (**d**) Section from Stano group highlighting the intense IL1βencircling blood vessels (arrow) along with cardiac myofibers (arrowhead) with significant difference (*P* ≤ 0.001) from C, EP, & PRP groups. (**e**) SEP group demonstrating few IL1β encircling blood vessels (arrow) along with cardiac myofibers (arrowhead) with significant difference (*P* ≤ 0.001) from C, EP, PRP & Stano groups. (**f**) SPRP group showing moderate IL1β encircling blood vessels (arrow) along with cardiac myofibers (arrowhead) with significant difference (*P* ≤ 0.001) from C, EP, PRP, Stano & SEP groups. Scale Bar = 50 μm. Area%; Values expressed as Mean ± SD (Area %), Different superscripts (**a**, **b**, and **c**) indicate significant difference at *p*-value ≤ 0.001

**Fig. 6:**
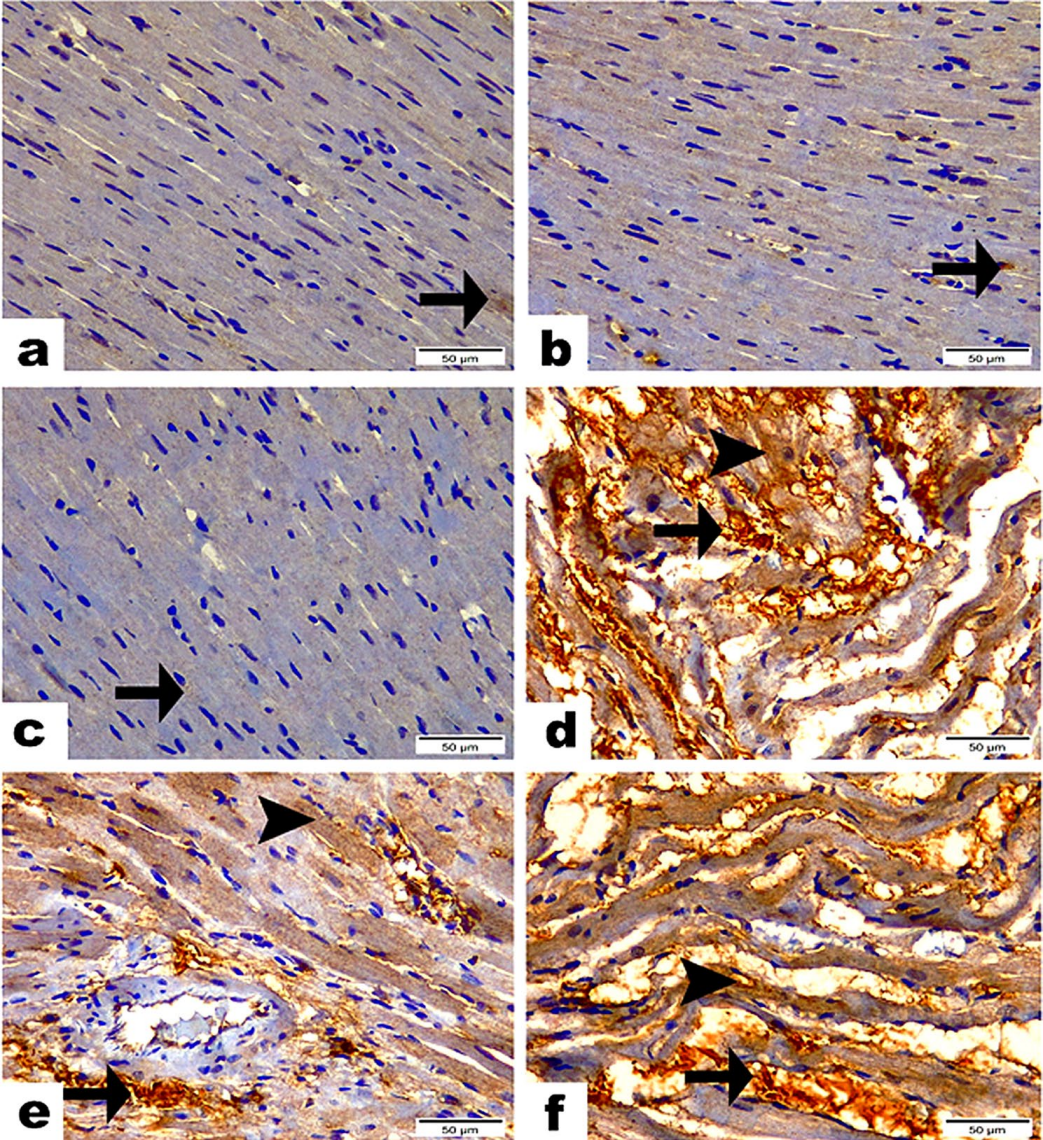
Photomicrographs demonstrated the reactivity of NF-κB/P65 along heart tissue among studied groups. Sections from C group (**a**), EP group (**b**), & PRP group (**c**) representing scares cytoplasmic NF-κB/P65 reactivity along cardiac myocytes (arrows) with non- significance between them. (**d**) Stano group highlighting the intense cytoplasmic (arrow) as well as nuclear (arrowhead) reactivity of NF-kB along cardiac myofibers and myocytes with significant difference (*P* ≤ 0.001) from C, EP, & PRP groups. (**e**) SEP group showing few cytoplasmic (arrow) as well as nuclear (arrowhead) reactivity of NF-κB/P65 along cardiac myofibers and myocytes with significant difference (*P* ≤ 0.001) from C, EP, PRP & Stano groups. (**f**) SPRP group revealing moderate cytoplasmic (arrow) as well as nuclear (arrowhead) reactivity of NF-kB along cardiac myofibers and myocytes with significant difference (*P* ≤ 0.001) from C, EP, PRP, Stano and SEP groups. Scale Bar = 50 μm. Area%; Values expressed as Mean ± SD (Area %), Different superscripts (**a**, **b**, and **c**) indicate significant difference at *p*-value ≤ 0.001

In the C group (G1) and the EP (G2) and PRP (G3) groups, the reactivity for IL-1β and NF-κB/P65 was either minimal or absent. No significant differences were found among these groups regarding these inflammatory markers. Rats treated with Stano displayed a significant increase in IL-1β and NF-κB/P65 levels, evidenced by strong positive brown staining in cardiomyocytes. This increase was statistically significant (*P* < 0.001) compared to the C groups, (Figs. 5 and 6). In contrast, the SEP (G5) and SPRP (G6), showed mild to moderate immunological responses for IL-1β and NF-κB/P65, respectively (Table 4). These responses were significantly different (*P* < 0.001) from both the C and Stano-only groups. Intriguingly, The EP and PRP therapies seem to significantly reduce the inflammatory response induced by Stano. Regarding to the quantitative scoring for immunohistochemistry results “Area Percentage”, the Stano (G4) treated rats displayed the high score, however the using EP and PRP therapies significantly improved the inflammation induced by S by expressing moderate to minimal IHC stain.

**Table 4:** Results of Caspase 3, IL1β, and NF-κB/P65 markers

	Control	EP	PRP	Stano	SEP	SPRP
Caspase 3	2.003 ± 0.563	1.837 ± 0.366	2.046 ± 0.933	45.759 ± 2.284a	17.989 ± 1.649c	27.488 ± 2.356b
IL1β	3.435 ± 0.606	3.463 ± 0.689	2.811 ± 0.9432	67.186 ± 2.066a	25.479 ± 1.814c	45.694 ± 3.57b
NF-κB/P65	4.259 ± 0.683	3.327 ± 0.686	2.639 ± 0.796	58.269 ± 1.663a	17.913 ± 3.359c	36.892 ± 2.968b

Values expressed as Mean ± SD. Different superscripts (a, b, c and d indicate significant difference at *p*-value ≤ 0.001

### Apoptosis assay (Caspase-3)

**Fig. 7:**
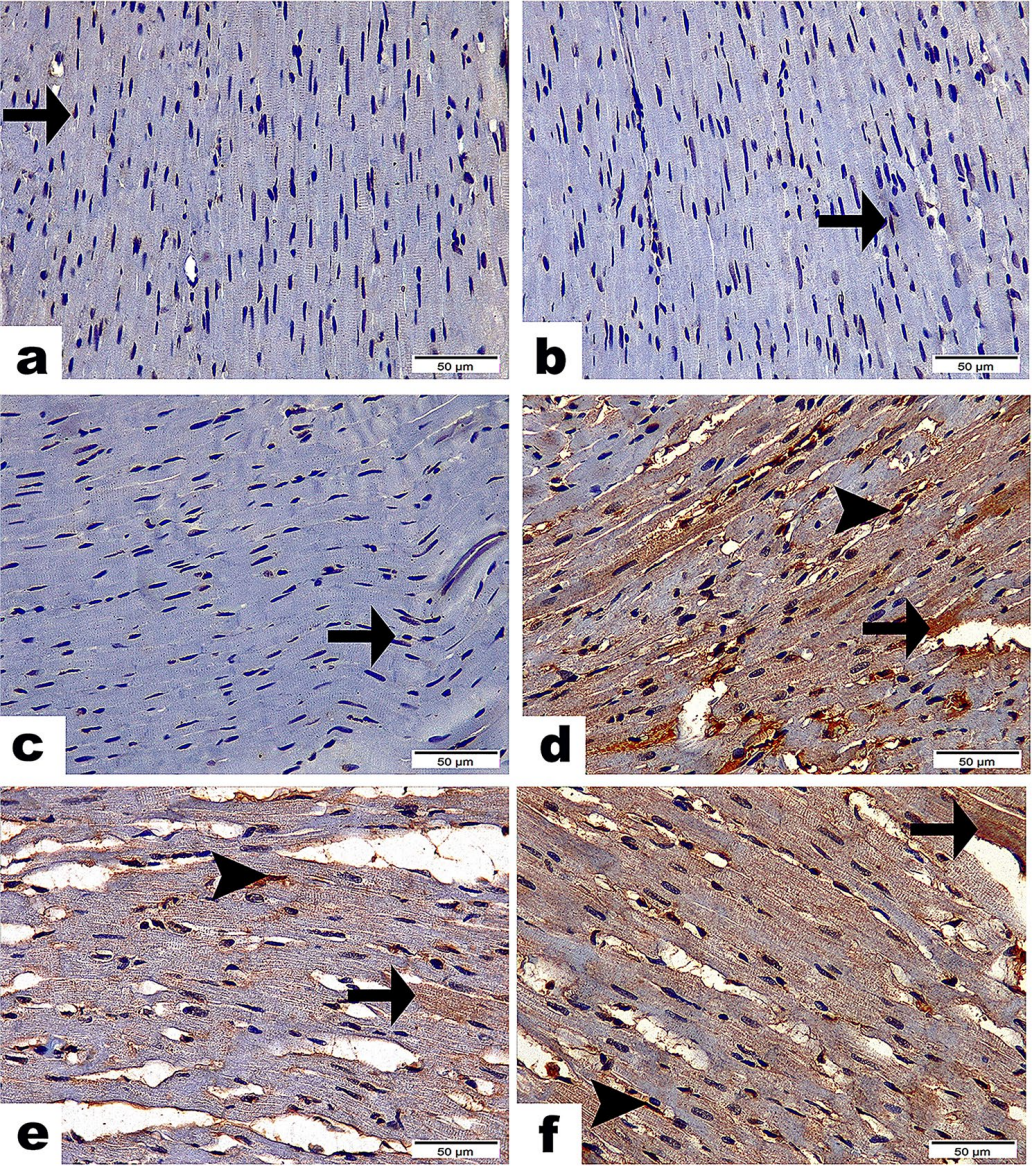
Photomicrographs demonstrated the reactivity of Caspase 3 along heart tissue among studied groups. Sections from C group (**a**), EP group (**b**), & PRP group (**c**) Emphasizing scare cytoplasmic reactivity along cardiac myocytes (arrows) with non-significance between them. (**d**) Stano group highlighting the intense cytoplasmic (arrow) as well as nuclear (arrowhead) reactivity of Caspase 3 along cardiac myofibers and myocytes with significant difference (*P* ≤ 0.001) from C, EP, & PRP groups. (**e**) SEP group marking few cytoplasmic (arrow) as well as nuclear (arrowhead) reactivity of Caspase 3 along cardiac myofibers and myocytes with significant difference (*P* ≤ 0.001) from C, EP, PRP & Stano groups. (**f**) SPRP group disclosing moderate cytoplasmic (arrow) as well as nuclear (arrowhead) reactivity of Caspase 3 along cardiac myofibers and myocytes with significant difference (*P* ≤ 0.001) from C, EP, PRP, Stano & Stano + EP groups. Scale Bar = 50 μm. Area%; Values expressed as Mean ± SD (Area %), Different superscripts (**a**, **b**, and **c**) indicate significant difference at *p*-value ≤ 0.001

The investigation into caspase-3 expression provides important insights regarding its involvement in apoptosis across various treatment groups. The C group (Fig. 7a), along with the EP group (Fig. 7b) and the PRP group (Fig. 7c), displayed negative staining, which indicates an absence of caspase-3 expression and suggests minimal apoptotic activity in these conditions. Conversely, the Stano group (d) exhibited a strong brown positive reaction for caspase-3, signifying a notable increase in apoptotic activity (*P* < 0.001). This finding implies that Stano may promote apoptosis in cardiac myofibers and myocytes. The administration of EP in SEP (G5) resulted in a weak response regarding caspase-3 expression (Fig. 7e), while the PRPS (G6) showed a moderate response (Fig. 7f). Both EP and PRP treatments demonstrated a significant difference when compared to the C and Stano-only groups (*P* < 0.001). Importantly, administration of EP and PRP led to a significant decrease in caspase-3 immunostaining (*P* < 0.001), (Table 4). Among these treatments, EP proved to be the most effective, surpassing PRP in its ability to lower caspase-3 levels.

## Discussion

Stano is a synthetic ASS derived from dihydrotestosterone (DHT) and has various properties either in medical or non-medical settings. However, its validation in medical uses, such as hereditary and angioedema treatment, its potential for misuse particularly by athletes in gyms and sports raises serious health concerns, especially regarding cardiovascular effects [8, 12]. Our lab results indicate that rats that received Stano expressed significant cardiac hypertrophy, characterized by reduced LV chamber sizes during diastole and systole, thickening of the septum with cardiac dysfunction and that was in line with numerous studies that have highlighted the negative cardiovascular impacts associated the misuse of the Stano [5, 53, 54].

The excessive misuse of ASS such as Stano weakens the mitochondrial respiratory chain complex activity, leading to an over-release of reactive oxygen species (ROS). This imbalance contributes to enhanced oxidative stress and cellular damage, which is documented here in our study by a noteworthy decline in CAT and GSH levels [55]. CAT plays an essential role in protecting and maintaining cells from oxidative injury by neutralization of ROS, as the overproduction of ROS results in significant harm to cellular structures, particularly during periods of oxidative stress [47]. CAT depletion can cause an upregulation in the level of hydroxyl radicals via Fenton’s reaction, leading to excessive damage to lipids and other cellular elements by the formation of MDA, a toxic byproduct linked to cellular injury and inflammation [56]. MDA serves as a critical vital indicator of oxidative damage, resulting primarily from the breakdown of the lipid, which can further lead to considerable disruption of the mitochondrial membrane, along with alterations in cellular proteins and DNA integrity [57, 58]. Our results findings have shown that the use of Stano markedly increases MDA levels, particularly in cardiac tissue. The elevation in MDA levels is closely linked to the extensive production of free radicals, which can surpass the antioxidant defenses of the body and adversely affect the function and morphology of heart tissue [47].

Elevation of the MDA levels resulted in the disruption of the permeability and integrity of the cell membrane, leading to the release of cardiac enzymes, specifically CK-MB and LDH, into the bloodstream, causing elevation of levels of this enzyme in the blood [59, 60]. Our findings are consistent with previous studies that demonstrated a strong positive correlation between CK-MB levels and MDA accumulation in heart tissue [4].

Uprising levels of ROS can trigger pro-inflammatory gene expression, leading to the increased release of inflammatory cytokines and so worsening the inflammation in the cardiac tissues [47, 61]. According to our findings, Stano significantly elevates the levels of pro-inflammatory cytokines, including IL-1β and NF-κB/P65, in the cardiac tissue. NF-κB/P65 is a redox essential transcription factor that is regulated by redox states. It remains inactive in the cytoplasm under typical conditions, tethered to inhibitor proteins known as IκBs. When activated and triggered by various stimuli such as mediators or oxidative stress, these IκBs are subsequently phosphorylated and then degraded [58, 62]. This process allows the movement of the NF-κB/P65 into the nucleus, which further initiates the expression of cytokines such as IL-1β that drive inflammatory responses [58]. This increase is further evidenced by enhanced immunostaining for these markers, indicating a pronounced inflammatory response in the cardiac tissue along with enhanced pathological alterations following S exposure.

Stano can enhance ventricular dysfunction and remodeling by altering the phosphorylation of phospholamban in animal models [12, 63]. These alterations may impair cardiac relaxation and contractility and contribute to structural modifications in cardiac tissue, exacerbated by oxidative stress. Furthermore, these findings are in line with our histopathological assessments, which revealed myocardial fibers disorganization, focal hypertrophy, amyloidosis, degenerative and necrotic changes within the cardiomyocytes that mirror the oxidative stress effects induced by Stano treatment [64]. A notable increase in the mean area percentage of collagen fibers in heart muscle following Stano administration using the Mallory Trichrome and Sirius Red immunostaining. This finding reveals a pronounced presence of fibrosis, supporting the previous observations by [14] about how collagen fiber accumulation and extracellular matrix remodeling can contribute to heart failure. The research emphasizes that Stano may promote fibrosis through mechanisms such as oxidative stress and cell death, evidenced by increased levels of cardiac fibrotic markers. This is correlated with our existing research, suggesting that AAS can lead to subtle cardiovascular changes, including an increase in the left ventricular mass due to collagen buildup. In contrast, cardiac amyloidosis is marked by the presence of numerous amyloid deposits within heart tissue, which is a separate condition linked to restrictive cardiomyopathy [65]. These alterations are linked to both oxidative stress (rise in MDA levels), along the corresponding histological alterations, highlighting the harmful impact of increased oxidative stress on the integrity and functionality of cardiac tissue and emphasizing the complex relationship between these factors in mediating cardiotoxic effects.

Mitochondrial oxidative stress plays a pivotal role in activating numerous cell signaling pathways, particularly those that lead to apoptosis, a form of programmed cell death governed by intricate regulatory mechanisms [66]. Caspase-3 is often referred to as an “executioner” caspase because it is one of the final steps in the apoptotic cascade. Upon activation, it cleaves numerous substrates that lead to characteristic morphological changes associated with apoptosis, such as chromatin condensation, DNA fragmentation, cell shrinkage, and membrane blebbing [67]. Moreover, caspase-3 is not solely a mediator of cell death but also plays crucial roles in supporting normal physiological functions and maintaining cellular homeostasis [66]. Our lab investigation documented that Stano treatment notably enhances apoptosis in myocardial tissue, evidenced by a significant rise in caspase-3 expression in the Stano treated group concerning the negative control group. This result aligns with the previously established understanding of how increased apoptosis contributes to cardiac tissue death and damage, potentially leading to adverse outcomes linked to ASS use, such as cardiomyopathy and other heart-related disorders.

EP, an SGLT2 inhibitor, has gained recognition for its dual role as an antihyperglycemic agent besides its renoprotective and cardioprotective properties, particularly in patients with type 2 diabetes mellitus (T2DM) who suffered existing cardiovascular dysfunction [68]. Studies indicated that this medication significantly reduced cardiovascular risks and enhanced various cardiac functions [69]. That is in accordance with our current work that shows that EP lowers oxidative stress markers (MDA) by lowering reactive oxygen species (ROS) production and enhancing mitochondrial energy efficiency by lowering the CK-MB and LDH levels [70], which referred a decline in myocardial damage [71]. It also boosts the antioxidant mechanism by upregulation of the GSH and CAT levels. This reduction in oxidative stress is crucial for mitigating myocardial damage, especially in cases of diabetic cardiomyopathy with keeping cardiac stability [72]. Additionally, EP improves left ventricular (LV) mass and ejection fraction key indicators of heart health. Notably, it has been observed to reverse LV mass enlargement and enhance functional capacity as assessed by various clinical tests in groups that received EP after administration of the Stano [73, 74]. Furthermore, EP reduces inflammatory markers like IL-1β and NF-κB/P65 pathways, which are associated with cardiac remodeling and injury [75]. Intriguingly, based on the histological findings, the EP supports tissue regeneration and the rearrangement of cardiomyocytes by decreasing collagen fiber buildup. It potentially lower the risk of severe heart failure episodes [19], associated with structural changes in the heart [76].

In the realm of regenerative medicine, PRP is emerging as a promising therapeutic option, particularly for cardiac conditions [25]. Our research has highlighted its cardioprotective effects, which are vital for treating various heart ailments. Platelets are vital components of the blood, primarily responsible for clotting and facilitating tissue repair. They contain three main types of secretory granules including α-granules, dense granules, and lysosomes that are rich in adhesive proteins, glycoproteins, and factors crucial for blood clotting and inflammation [77].

As PRP has been documented to sustain systolic function and reduce heart hypertrophy caused by Stano with remarkable improvements in cardiac performance [78]. Furthermore, treatment with PRP resulted in a decrease in CK-MB and LDH levels, suggesting a reduction in injury and damage to the cardiac tissue [79]. This finding supports the notion that PRP can diminish oxidative stress markers and promote overall cardiac healing. Additionally, PRP exhibits anti-inflammatory properties with a reduction of apoptosis that helps manage the inflammatory response and cell apoptosis following myocardial injury by modulating inflammatory markers as seen in our study, PRP can create a more favorable environment for healing and regeneration. The activated platelets release platelet microparticles (PMPs), which play a crucial role in regulating inflammation and immune responses [80]. PMPs have anti-inflammatory properties that help reduce the activation of macrophages and dendritic cells and inhibit the production of interleukin-17 (IL-17) by regulatory T cells, which is important for resolving inflammation in tissues [81]. Additionally, PMPs can change the polarization of monocytes and macrophages towards less reactive states. This shift is important for minimizing tissue damage and facilitating healing during inflammatory responses [82].

The positive effects of PRP are largely due to its high concentration of growth factors, including VEGF, transforming growth factor-beta (TGF-β), and platelet-derived growth factor (PDGF). These factors alleviate inflammation, encourage angiogenesis, and aid in tissue regeneration and repair [25, 27]. While PRP shows significant potential, its rate of cardiac tissue architecture improvement indicators may be slower when compared to the EP treatment. This slower response may be attributed to the time necessary for inflammatory regions to heal and regenerate properly [25, 27].

Both cardiovascular protective effects of EP and PRP in cardiovascular diseases share some overlapping mechanisms even though they are two different therapies through different mechanisms and modes of action. They are capable of supplementing one another in cardiovascular disease through the induction of anti-inflammatory effects. EP reduces systemic inflammation, while PRP Cs cardiac tissue inflammation [79]. PRP directly induces angiogenesis by growth factors [79], but EP may indirectly help the process by overall cardiovascular health improvement. EP has also been shown to reduce adverse cardiac remodeling [83], and PRP may also help with this effect because it is a regenerative treatment [79]. Both of these treatments possess the ability to neutralize oxidative stress, which is a significant component in the advancement of cardiovascular disease [79]. EP supports the energy balance of the heart by increasing the production of ATP [83]. PRP may complement this by providing growth factors that optimize cellular metabolism and regeneration [79]. Such theoretical synergies are intriguing but, to our knowledge, have not been examined directly in human studies comparing or combining EP and PRP in cardiovascular disorders. Additional research is required to examine the possible additive or synergistic interaction of these interventions in the control of cardiovascular disease.

## Conclusion

Our study elucidated the negative cardiac outcomes associated with the Stano administration, an AAS, as it can lead to ventricular dysfunction which contributes to cardiac hypertrophy and remodeling. This steroid also upregulates oxidative stress and induces fibrotic and pathological changes in the cardiac tissue, enhancing the risk of severe cardiovascular incidents, such as heart attacks. The remarkable cardioprotective benefits of EP and PRP highlight the mitigation effect of both substances against the Stano. PRP relieved the adverse myocardial injuries linked to Stano use through potential improvements in the heart antioxidant defense mechanism by lowering the MDA levels and increasing the CAT and GSH levels, along with downregulation of the inflammatory cytokines and apoptotic death. The steroid’s capacity to cause substantial cardiac dysfunction underscores the importance of thorough monitoring and risk evaluation for those considering its use. Ongoing research is crucial to further elucidate the mechanisms by which PRP aids cardiac health and to refine its clinical applications, ultimately aiming to optimize patient outcomes in regenerative medicine. 

## Data Availability

No datasets were generated or analysed during the current study.

## Abbreviations

ABC: Avidin- biotin peroxidase
CK-MB: Creatine kinase myocardial band
CAT: Catalase
EP: Empagliflozin
FS: Fractional shortening
GSH: Reduced glutathione
IL1β: Interleukin-1β
IVS: Interventricular septal thickness
LDH: Lactate dehydrogenase
LVID: Left ventricular internal diameter
LVW: Left ventricular posterior wall
MDA: Malondialdehyde
NF-κB/P65: Nuclear factor Kappa-B/p65
PBS: Phosphate buffer saline
PRP: Platelet-Rich Plasma
ROS: Reactive oxygen species
Stano: Stano
